# Brazilian research on eating disorders: historical factors, research management, and consequences

**DOI:** 10.1590/1806-9282.20241210

**Published:** 2024-12-02

**Authors:** Jônatas de Oliveira

**Affiliations:** 1 Universidade de São Paulo, FMUSP, Faculdade de Medicina – São Paulo (SP), Brazil.; 2 Instituto de Pesquisa em Comportamento – São Paulo (SP), Brazil.

Dear Editor,

A recent Brazilian study provided an analysis of the profile of patients with eating disorders (EDs) treated by the first specialized Brazilian ED service, the Support Group for Eating Disorders (Grupo de Assistência em Transtornos Alimentares—GRATA), which was founded in 1982. The study included a thorough analysis of patients who were followed until 2019^1^. GRATA effectively illustrated the potential of a computerized data system to be utilized and reported by other health services, thereby establishing a support network for Brazilian research and facilitating the comprehension of patient pathways and profiles. Furthermore, it prompted us to reexamine historical data and gain insight into the national trajectory and landscape, with a particular focus on the southeastern region^1^. This observation has prompted discussions about health inequities and patient pathways^2^.

While developing a research project to contextualize the Brazilian national landscape, I realized I was frequently looking beyond Brazil, concentrating on the etiological models of Northern countries and their influence on research strategies, teaching, and the understanding of EDs within Brazil. However, this approach is not the most appropriate. An examination of the historical development, evolution, and direction of research in EDs in Brazil can provide insights into the current state of the field. In my subsequent readings, I encountered:

*… I attended the first patient with anorexia nervosa in 1969. She was a patient around 15 years old who was periodically hospitalized … What struck me was the "illogical" nature of someone voluntarily stopping to eat (Dr. José Ernesto dos Santos)* ^3^.

From Santos’ pioneering work:

*The need for the formation of a multidisciplinary team to care for these patients also arose from a simple observation. However, to reach this conclusion, we needed to adopt the uncomfortable stance of relinquishing our omnipotent and omniscient perspectives to see that when a patient stops eating, without an organic cause, it is not due to a conscious personal desire but due to other causes. Traditional and isolated nutritional guidance does not have the desired efficacy and efficiency for their treatment (Dr. José Ernesto dos Santos)* ^3^.

The earliest citation of a clinical description of EDs in Brazil is attributed to GRATA in 1986. Subsequently, in Rio de Janeiro, the Grupo de Obesidade e Transtornos Alimentares emerged following a unification in 1991^4^.

In 1991 … the Psychoneuroendocrinology Unit was created, under the coordination of psychiatrist Dr. José Carlos Appolinário and the endocrinologist Walmir Coutinho, … (cf. institution's website). In 1992 … Assistance and research in binge eating began, soon expanding to cover the entire spectrum of eating disorders^4^.

In a third and last moment, AMBULIM was proposed in 1992 by Táki Athanássios Cordás:

"When there was no specialized center for the treatment of EDs in Brazil" (AMBULIM, no date)^5^.

It is both surprising and troubling that research on EDs in Brazil remains so scarce, particularly given the prevalence of these disorders in the country. The fact that a systematic review on this topic could be perceived as both innovative and feasible serves to underscore the substantial research gap that currently exists. During my doctoral studies, I conducted an exhaustive review of all published research involving Brazilian individuals with EDs and identified fewer than 100 studies. In 2023, fewer than 10 studies were published. In any simple search of the databases we routinely use, the lack of national studies is glaringly evident. The fact that this topic is novel and is being addressed at the doctoral level already demonstrates how far behind we are compared to the rest of the world. It would be impossible to conduct a review that encompasses all the studies conducted in the United States on populations with EDs.

In the Brazilian context, effectively addressing EDs requires proactive engagement of human resources. We still do not have integrated information technology, data systems, and data collection processes connected to the national health systems. We rely on postgraduate students to collect data and publish their findings to gain an understanding of the broader picture, albeit with many gaps. The lack of shared information systems often forces researchers to rely on the work of undergraduate students who are selected to work with EDs, though not all are given this opportunity. Meanwhile, other regions of the world have made more substantial progress in researching severe EDs.

After conducting a thorough search (August 2024) in the *International Journal of Eating Disorders*, I identified 18 publications by cross-referencing the terms (eating disorder, anorexia nervosa, bulimia nervosa, binge eating disorder, pica, rumination disorder, and avoidant/restrictive food intake disorder). Among these, there are three Brazilian publications from 2023, none of which were conducted with an ED sample. Additionally, upon reviewing the remaining articles, I did not find any original studies with data from Brazilian individuals with EDs.

The construction of a robust and shared database can thus make a significant contribution to advancing the understanding and treatment of ED on a nationwide scale, including the treatment withdrawal numbers, and acting on measures to prevent the abandonment, such as creating better humanized protocols and working hard for their democratization across the country. However, this depends on data management practices and the assignment of research roles in clinical practice, as these elements are not necessarily interconnected.

In the absence of research, there can be no knowledge. Without knowledge, it is impossible to create protocols or to provide interdisciplinary education or training in mental health. It is therefore crucial to comprehend the incorporation of disciplines in the undergraduate and postgraduate curricula for medical practitioners and other health professionals engaged in the direct treatment of EDs throughout Brazil. Currently, studies and programs pertaining to EDs are predominantly concentrated in the country's southeastern region. Advancements in this field are contingent upon research. However, national research in this area is significantly delayed and progresses at a slow pace.
